# Hot on RAD51C: structure and functions of RAD51C‐XRCC3

**DOI:** 10.1002/1878-0261.13518

**Published:** 2023-09-11

**Authors:** Barnabas Szakal, Dana Branzei

**Affiliations:** ^1^ IFOM ETS, The AIRC Institute of Molecular Oncology Milan Italy; ^2^ Istituto di Genetica Molecolare, Consiglio Nazionale delle Ricerche (IGM‐CNR) Pavia Italy

**Keywords:** DNA binding, Fork protection, Fork restart, RAD51 filament capping, RAD51 paralogs, RAD51‐XRCC3

## Abstract

A new study by Longo, Roy et al. has solved the structure of the RAD51C‐XRCC3 (CX3) heterodimer with a bound ATP analog, identifying two main structural interfaces and defining separable replication fork stability roles. One function relates to the ability of RAD51C to bind and assemble CX3 on nascent DNA, with an impact on the ability of forks to restart upon replication stress. The other relates to effective CX3 heterodimer formation, required for 5′ RAD51 filament capping, with effects on RAD51 filament disassembly, fork protection and influencing the persistence of reversed forks.

## Graphical abstract

A new study by Longo, Roy et al. has solved the structure of the RAD51C‐XRCC3 (CX3) heterodimer with bound ATP analog, identifying two main structural interfaces. Studies of cancer mutations of previously unknown significance in the two interfaces define separable replication fork stability roles required for fork protection and restart.

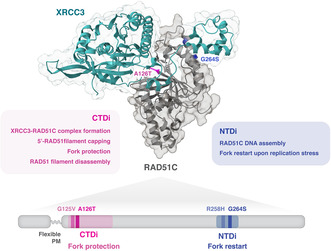

**Abbreviations**
ap: *Alvinella pompejana*
BCDX2: RAD51B‐RAD51C‐RAD51D‐XRCC2
CTD: C‐terminal domain
CTDi: C‐terminal domain interface
CX3: RAD51C‐XRCC3
hCX3: human CX3
HDR: homology‐directed repair
NTD: N‐terminal domain
NTDi: N‐terminal domain interface
PM: polymerization motif
SIRF: *In situ* protein interaction with nascent DNA Replication Forks
VUS: variants of unknown significance

RAD51C has long intrigued cancer biologists. Comprehensive analyses of breast and ovarian cancer pedigrees and patients with Fanconi anemia, a rare disorder characterized by hematological and developmental defects, pinpointed RAD51C as a cancer predisposition gene [1, 2]. This finding was independently confirmed by sequencing of cancer genomes, where variants of unknown significance (VUS) mapping throughout the protein sequence could not be ignored [3].

Functional inquiries on RAD51C have been going on for decades. This is due to its role in interacting and assisting RAD51's recombinase function [4], central to our understanding of homology‐directed repair (HDR) of double‐strand breaks and homologous recombination‐mediated replication initiated by and potentially helping replication problems [5, 6]. RAD51C is one of the central members of the RAD51 paralog family of proteins, sharing similarity with RAD51 and having critical functions in RAD51‐mediated reactions. Similar to RAD51, Walker A/B ATP binding motifs are present in RAD51C, in addition to a putative Holliday Junction resolvase activity residing within its N‐terminal region [7]. However, the precise function of RAD51C has remained enigmatic.

Two main complexes of RAD51 paralogs are known, with RAD51C being common to both [8]. In one of them, RAD51C binds XRCC3 to form a heterodimer known as CX3 [9], and in the other one, RAD51B combines subsequently with RAD51D‐XRCC2 to yield a hetero‐tetramer, BCDX2, where all paralogs except RAD51B are considered essential. The paralogs have incompletely understood functions in RAD51 loading and filament stabilization, resembling those of BRCA2 [10], and further functions in mitigating replication stress, including roles in replication fork protection and restart [5, 11]. These functions of RAD51 and the paralogs are broadly divided into HDR and replication stress response. However, mechanistically, they likely converge into aspects of RAD51 loading, assembly and disassembly that are critical for the ability of cells to complete replication without accumulating DNA damage that can predispose the organisms to cancer. However, some key questions remain: What are these functions and why do certain RAD51C mutations have a much more severe phenotype than others?

Important insights on these topics came recently through the teamwork of several groups, combining structure elucidation of the CX3 complex with functional studies of specific cancer variants [12]. Ingenuity was required at several steps during this work. The first technical challenge was related to the ability to purify an active complex, a task that the team could successfully accomplish using thermostable and highly conserved RAD51C and XRCC3 proteins from *Alvinella pompejana* (ap), an extreme metazoan living in deep‐sea waters. Leaving out a small, resolution limiting N‐terminal domain (NTD) of RAD51C and using a non‐hydrolyzable ATP mimic bound in the active sites of both RAD51C and XRCC3, the authors solved high‐resolution crystal structures of the CX3 heterodimer. This revealed extensive contacts between the RAD51C C‐terminal domain (CTD) and XRCC3 NTD and CTD, with these subdomains connected by a linker polymerization motif (PM) of XRCC3. Although similar to RAD51 structures in terms of ATP binding [13], the CX3 structure revealed a unique positioning of the XRCC3 NTD, rotated about 90 degrees compared to RAD51‐RAD51 interactions, enabled by the unique XRCC3 linker PM. This structure resembles the one of a clamp with an NTD interface (NTDi) established by the XRCC3 NTD facing a surface of RAD51C, and a CTD interface (CTDi), established by the XRCC3 and RAD51C proximal CTD domains around the ATP‐binding sites. Using several approaches and algorithms, the authors infer that the obtained structure is generally representative of the evolutionarily conserved CX3 complex.

Relying on the 3D representation of the CX3 heterodimer, the authors noticed that the cancer and patient mutations can be largely segregated in the two distinct interaction regions, CTDi and NTDi [1, 2, 14]. Moving on, the authors aimed to probe the significance of mutations that previously remained obscure, due to a lack of overt defects in HRD proficiency [1, 2]. They chose to showcase mutations proximal in 3D to ones previously demonstrated to be defective in HDR. The approach taken, however, was not trivial as RAD51C is essential. While previous studies used overexpression of RAD51C variants in cells with RAD51C knockdown, the authors decided to bypass the potential confounding effects of that approach. Instead, authors used CRISPR‐Cas9 edited haploid HAP1 cells with endogenously expressed RAD51C selected variants, affecting either CTDi or the NTDi. They chose A126T to inquire about the CTDi function, as this residue neighbors a deleterious G125V mutation, proximal to both the ATP binding site and an XRCC3 binding interface [15]. Regarding the NTDi, they chose the G264S mutation, located on an alpha‐helical patch in proximity to the previously characterized deleterious R258H mutation [1]. The HAP1 cells expressing patient mutations were viable and lacked HDR defects, but impaired proliferation and showed increased micronuclei, suggesting unresolved replication stress.

What is the nature of the persisting replication stress? A126 is located on the P‐loop required to bind the ATP phosphate and is capable of disrupting the CX3 interface. Indeed, while the G125V mutation greatly impacts the structure and causes loss of interaction with XRCC3 as observed by two‐hybrid, the A126T mutation reduces the interaction with XRCC3 upon replication stress, as measured by proximity ligation assays or by co‐immunoprecipitation. While the G264S mutation does not destabilize the interaction with XRCC3, it greatly reduced the interaction of RAD51C with nascent DNA at stalled forks as revealed by *in situ* protein interaction with nascent DNA replication forks (SIRF). Besides this separation of function, the authors attempted to predict hCX3‐RAD51 complexes using AlphaFold2. In this way, they realized that XRCC3, but not RAD51C, interacts with RAD51 [4] and inferred that CX3 blocks RAD51 filament growth and disassembly on the 5′ end. Further using SIRF assays at different times upon exposure to replication stress, they showed that A126T causes faster RAD51 disassembly from stalled forks, while the G264S mutation prevents efficient assembly of RAD51 upon replication stress. These results further correlate with A126T having strong defects in fork protection, but not fork reversal, which appears increased, and with G264S having severe defects in fork restart.

Thus, CX3 plays important hitherto unappreciated roles during DNA replication stress, using different interfaces to facilitate fork restart or to protect forks and resolve emerging reversed forks. This work creates the foundation to study the multiple CX3 functions relevant to cancer biology at the nexus with replication stress response.

## Conflict of interest

The authors declare no conflict of interest.
